# Efficacy of a novel topical combination of esafoxolaner, eprinomectin and praziquantel against *Amblyomma americanum* in cats

**DOI:** 10.1051/parasite/2021021

**Published:** 2021-04-02

**Authors:** Joe Prullage, Christine Baker, Abdelmoneim Mansour, Scott McCall, David Young, Eric Tielemans

**Affiliations:** 1 Boehringer-Ingelheim Animal Health, Missouri Research Center 6498 Jade Rd. Fulton MO 65251 USA; 2 Boehringer-Ingelheim Animal Health 1730 Olympic Drive Athens GA 30601 USA; 3 TRS Labs Inc. 215 Paradise Blvd Athens GA 30607-1151 USA; 4 Young Veterinary Research Services 7243 East Avenue Turlock CA 95380-9124 USA; 5 Boehringer-Ingelheim Animal Health 29 avenue Tony Garnier 69007 Lyon France

**Keywords:** Cat, Esafoxolaner, Tick, Efficacy, *Amblyomma americanum*

## Abstract

Esafoxolaner, a purified enantiomer of afoxolaner with insecticidal and acaricidal properties, is combined with eprinomectin and praziquantel in NexGard^®^ Combo, a novel topical endectoparasiticide product for cats. The efficacy of this novel formulation was assessed in two experimental studies against induced infestations with *Amblyomma americanum*, a tick species of major importance, highly prevalent in a large southeastern quarter of the United States. In each study, 10 cats were randomly allocated to a placebo control group and 10 cats to a novel formulation treated group. Infested cats were treated topically once at the minimum recommended dose. Both studies were designed to test curative efficacy on existing infestation, 72 h after treatment, and to test preventive efficacy, 72 h after subsequent weekly (Study #1) or fortnightly (Study #2) infestations for one month. For each infestation, all cats were infested with 50 unfed adult *A. americanum*. At each tick count, in both studies, at least 8 in 10 placebo control cats were infested with 13 (26%) or more live ticks, demonstrating adequate infestation throughout the studies. Curative efficacy of the novel formulation was 99% in both studies; preventive efficacy was 92% and 100% for at least one month.

## Abstract (fr)

L’esafoxolaner, un énantiomère purifié d’afoxolaner aux propriétés insecticides et acaricides, est associé à l’éprinomectine et au praziquantel dans NexGard^®^ Combo, un nouvel endectoparasiticide topique pour chats. L’efficacité de cette nouvelle formulation a été évaluée dans deux études expérimentales contre les infestations induites par *Amblyomma americanum*, une espèce de tique d’importance majeure, très répandue dans un grand quart sud-est des États-Unis. Dans chaque étude, dix chats ont été répartis au hasard dans un groupe témoin placebo et dix chats dans un groupe traité par une nouvelle formulation. Les chats infestés ont été traités une fois par voie topique à la dose minimale recommandée. Les deux études ont été conçues pour tester l’efficacité curative sur une infestation existante, 72 heures après le traitement, et pour tester l’efficacité préventive, 72 heures après des infestations hebdomadaires (étude n° 1) ou bimensuelles (étude n° 2) pendant un mois. Pour chaque infestation, tous les chats étaient infestés par 50 *A. americanum* adultes non nourris. À chaque décompte de tiques, dans les deux études, au moins 8 chats sur 10 du groupe témoin placebo étaient infestés de 13 (26 %) ou plus tiques vivantes, ce qui démontre une infestation adéquate tout au long des études. L’efficacité curative de la nouvelle formulation était de 99 % dans les deux études, l’efficacité préventive était de 92 % et 100 % pendant au moins un mois.

## Introduction

*Amblyomma americanum*, an Ixodid hard tick, also called the lone star tick, is widely distributed across the East, Center, South East and Midwest of the United States [1, 10, 14, 16, 21, 25, 35]. *Amblyomma americanum* is especially abundant in and around wooded areas populated by the white-tailed deer, considered to be the primary host for all developmental stages. Characterized by indiscriminant and aggressive feeding behavior, the lone star tick parasitizes a wide variety of medium and large mammals at all development stages, including cats, dogs, wildlife, livestock, humans, and ground dwelling birds like quails and wild turkeys [9, 19, 26, 30, 48]. *Amblyomma americanum* is seasonal and patterns of abundance vary across the United States. Generally, adults are prevalent on hosts during spring and decline through the summer, nymphs mainly appear in the spring and decline through the summer, and larvae appear in the summer and decline through fall [11, 16, 19].

The most common attachment sites of *A. americanum* in cats are the ventral, perianal and the tail areas [35]. Adult tick infestations can cause painful focal sores, but more discrete immature forms are responsible for an important proportion of cat infestations, which might contribute to an underestimation of the infestation level by owners and veterinarians [19].

Like all Ixodidae species, *A. americanum* is a vector of viral, bacterial and protozoan diseases affecting animals and also humans [4–6, 15, 27, 29, 38]. It is a public health concern in South East and Atlantic US states, where it is the tick species most frequently found attached to human beings [24, 25]. It transmits diseases such as ehrlichiosis (caused by *Ehrlichia* spp.), tularemia (caused by *Francisella tularensis*), and the southern tick-associated rash illness STARI (presumed to be caused by *Borrelia lonestari*) [2, 17, 24, 40]. However, it is not a competent vector of *Borrelia burgdorferi*, the agent of Lyme disease [39]. Importantly, *A. americanum* adults and nymphs are the main vector of *Cytauxzoon felis*, the most severe and often lethal tick-borne disease in cats [3, 7, 32, 36]. One study reported cytauxzoonosis transmission after exposure to adult *A. americanum* exceeding 36 h [41].

NexGard^®^ Combo, a novel topical combination of esafoxolaner, eprinomectin and praziquantel was developed to offer a wide parasiticide spectrum and integrated control of cat parasites. Esafoxolaner, a novel isoxazoline compound, is the active purified enantiomer of afoxolaner, the racemic mixture. Afoxolaner is commercially available as an oral acaricide and insecticide product for dogs and is efficacious against *A. americanum*, as a single active substance (NexGard^®^) or in combination with milbemycin oxime (NexGard Spectra^®^). Other isoxazoline compounds, such as sarolaner (in combination with selamectin) have been demonstrated to be efficacious against *A. americanum* in cats [20, 33, 37]. This article describes two studies performed to evaluate the efficacy of this novel formulation for the treatment and control of *A. americanum-*induced infestations in cats.

## Materials and methods

### Ethics

The study protocols were reviewed and approved by the Sponsor’s and local Institutional Animal Care and Use committees. Cats were managed and handled similarly and with due regard for their wellbeing.

### Study design

The studies were designed in accordance with the “World Association for the Advancement of Veterinary Parasitology (W.A.A.V.P.) guidelines for evaluating the efficacy of parasiticides for the treatment, prevention and control of flea and tick infestation on dogs and cats”. The studies were conducted in accordance with Good Clinical Practices as described in International Cooperation on Harmonization of Technical Requirements for Registration of Veterinary Medicinal Products (VICH) guideline GL9.

The two studies were conducted under a negative-controlled and randomized design, based on live *A. americanum* counts, 72 h after a pre-treatment infestation. The efficacy of the novel formulation was assessed by comparing live tick counts on the placebo control and the novel formulation-treated groups at identical weekly (Study #1) or fortnightly (Study #2) timepoints after treatment. All personnel collecting animal health and efficacy data were blinded to treatment assignments.

The studies were performed in two different licensed establishments in the United States, and had comparable designs. The relevant differences between both studies in terms of context, animals and timepoints are described in Table 1.

**Table 1:** Main differences between the two studies.

Study #	Location	Date	Animals			Schedules		
			Number and sex^1^	Age (months)	Bodyweight (kg)	Infestations	Treatment	Tick counts
1	California, USA	Sep–Oct 2017	10 M, 1 MC, 7 F, 3 FS	33–97	2.7–6.2	Days −2, 7, 14, 21, 30	Day 0	Days 3, 10, 17, 24, 33
2	Georgia, USA	May–Jul 2018	10 M, 10 F	7–8	2.5–5.6	Days −2, 14, 30	Day 0	Days 3, 17, 33

1 M = male, F = female, MC = male castrated, FS = female spayed.

### Animals and housing

Twenty purpose-bred, healthy Domestic Long/Short-hair cats were included in each study (Table 1). Cats were single housed during the study to avoid inter-animal treatment contamination, in an environmentally controlled facility. To allow the collection of dead ticks, cats were housed in enclosures that had mesh flooring above a plain collection pan.

### *Amblyomma americanum* isolates

In Study #1, laboratory maintained unfed adult *A. americanum* from a colony with new genetic material introduced nine months before (in January 2017) were used for the infestations. In Study #2, unfed adult *A. americanum*, wild-caught in Payne County, Oklahoma, USA in the spring of 2018 were used for the infestations. These ticks were not tested for pathogens.

### Treatment

Cats were treated once on Day 0. The treatment was applied topically on the skin, after parting the hair, on one spot in the midline of the neck between the base of the skull and the shoulder blades. Cats assigned to the placebo control group were administered mineral oil at 0.12 mL/kg, cats assigned to the novel formulation treated group were administered the novel formulation at the minimum recommended dose of 0.12 mL/kg, delivering 1.44 mg/kg esafoxolaner, 0.48 mg/kg eprinomectin and 10.0 mg/kg praziquantel.

### Health monitoring

Health observations were conducted by qualified personnel daily throughout the study and at hourly intervals for 4 h after treatment for the detection of any abnormal reaction.

### Tick infestations

In both studies, cats were infested 48 h before treatment to evaluate curative efficacy at 72 h after treatment, and then weekly or fortnightly 72 h before tick counts for preventive efficacy during the month following treatment (Table 1). In Study #1, cats were sedated, placed in an infestation crate, fitted with an Elizabethan collar and infested with 50 adult unfed *A. americanum* placed on the back and avoiding the treatment site. After a maximum of 4 h, cats were removed from the crates and the remaining free ticks placed back on the cat (the sedation was not reversed). The Elizabethan collar was left until the corresponding tick count procedure. In Study #2, the same procedure was followed, except that cats were infested in their cage and were not sedated.

### Tick counts

Tick counts were performed in a random order. Attached and unattached live and dead ticks were removed and counted approximately 72 h after treatment, and subsequent infestations (Table 1). Ticks were first detected visually and by parting and feeling through the cat’s hair with finger tips, and second by thorough combing of the haircoat using a fine-toothed flea comb. Each detected tick was immediately removed and observed for signs of viability or mortality. Protective clothing (e.g. gowns/coats, gloves, etc.) and combs were changed between each cat to prevent cross-contamination.

Dead ticks found in collection pans beneath wired mesh floor were counted and removed daily during the three days following each infestation, and were tallied to dead ticks found on cats after each infestation timepoint.

### Statistical analysis

To compute the percent efficacy, the arithmetic mean of the live tick counts was calculated per group and per infestation timepoint. At each timepoint, the percent efficacy of the novel formulation-treated group compared with the placebo control group was calculated using the formula [(*C* − *T*)/*C*] × 100, where *C* was the arithmetic mean for the control group and T the arithmetic mean for the treated group. Then, the log-count of the live ticks recovered from the treated group was compared to the log-count of the control group using an *F*-test adjusted for the allocation blocks at each timepoint separately on a two-sided 5% significance level.

The log-counts of dead ticks recovered from each cat and from the collection pan beneath the wired mesh floor after each infestation of the treated group were compared to the log-counts of the control group using an *F*-test adjusted for the allocation blocks at each timepoint separately on a two-sided 5% significance level. The MIXED procedure in SAS version 9.4 was used for both analyses, with group listed as a fixed effect and block listed as a random effect.

## Results

Table 2 illustrates the efficacy results.

**Table 2:** Efficacy of NexGard^®^ Combo against *Amblyomma americanum.*

Study	Group	*n*	Arithmetic means (AM) of live ticks and % efficacy 72 h after treatment or subsequent infestations					
				Day 3	Day 10	Day 17	Day 24	Day 33
#1	Control^1^	10	AM	18.8	24.0	23.7	22.7	25.0
	Novel formulation^2^	10	AM	0.1	0.4	1.7	1.3	2.1
			% efficacy^3^	99.5	98.1	93.0	94.1	91.6
			*p*-value^4^	<0.0001	<0.0001	<0.0001	<0.0001	<0.0001
			*p*-value dead ticks	<0.0001	<0.0001	<0.0001	<0.0001	<0.0001

#2	Control^1^	10(9)^5^	AM	27.0	–	32.1	–	26.3
	Novel formulation^2^	10	AM	0.2	–	0.0	–	0.0
			% efficacy^3^	99.3	–	100.0	–	100.0
			*p*-value^4^	<0.0001	<0.0001	<0.0001	<0.0001	<0.0001
			*P*-value Dead ticks	<0.0001	<0.0001	<0.0001	<0.0001	<0.0001

1 Cats were treated topically once on Day 0, each with 0.12 mL/kg mineral oil.

2 Cats were treated topically once on Day 0, each with 0.12 mL/kg of the novel formulation, providing 1.44 mg/kg esafoxolaner, 0.48 mg/kg eprinomectin, and 10.0 mg/kg praziquantel.

3 Percent efficacy = 100[(*C* − *T*)/*C*], where *T* and *C* are arithmetic means (AM) of the novel formulation and control groups, respectively.

4 *p*-value = two-sided probability value from analysis of variance using the MIXED model to compare the novel formulation group with the control group.

5 Ten cats on Day 3, then 9 cats on Days 17 and 33, because one cat died on Day 15 with acute cytauxzoonosis.

In Study #1, at all efficacy timepoints, an average of 22.8 live ticks was counted on the placebo control cats and the individual counts ranged from 5 to 40 live ticks. In Study #2, at all efficacy timepoints, an average of 28.4 live ticks was counted on the placebo control cats, and the individual counts ranged from 9 to 48 live ticks. In both studies and at each timepoint, at least 8 of the 10 control cats had 13 (26%) or more live ticks.

In both studies, curative efficacy of one application at the minimum dose of NexGard^®^ Combo on existing tick infestation 72 h after treatment was at least 99.3%. Preventive efficacy over the following four weeks, 72 hours after infestations was at least 91.6%, with a significant difference between the treated and control groups at all timepoints (*p* < 0.0001).

The numbers of dead ticks collected in the pan beneath each cat during the three days following each infestation, and from the cats at each tick count in both studies, were significantly higher in the NexGard^®^ Combo-treated groups than in the control groups at all timepoints (*p* < 0.0001).

No adverse reactions related to treatment were observed in either of the two studies. In Study #2, in which wild caught ticks were used, one cat from the placebo-treated group developed acute cytauxzoonosis 16 days after the pre-treatment infestation. The diagnosis was based on clinical signs (lethargy, fever, dehydration, weakness and hyperemic mucous membranes), hematology (pancytopenia), positive piroplasma *C. felis* blood smear, and histology (abundant organism-laden histocytes and erythrocytes in multiple organs).

## Discussion

The calculated efficacy results in these studies demonstrate high efficacy of NexGard^®^ Combo against *A. americanum* in cats, both curatively within 72 h of treatment application for existing infestations, and preventively within 72 h of new infestations occurring for at least one month after treatment. This result was attained using a vigorous tick population, as demonstrated by the retention of 13 or more ticks in 8 of 10 control cats, exceeding the WAAVP minimum adequate infestation requirement of 6 cats with 12 or more live ticks.

A recent study monitoring year-long ectoparasite infestations in free-roaming cats in the Central United States revealed an overall prevalence of flea and tick infestations of 87.2% and 18.7%, respectively. Among tick infested cats, *A. americanum* was the most prevalent species (65.9%), followed by *Ixodes scapularis* (32.5%), and *Dermacentor variabilis* (10.3%) [42]. Two recent studies characterizing ticks collected on cats in veterinary practices from the four main regions of the United States revealed that, depending on the region, *I. scapularis* or *A. americanum* were the most prevalent tick species [21, 35].

*Amblyomma americanum* is a major vector of *C. felis,* the agent of cytauxzoonosis, a life-threatening and often fatal protozoan disease of cats [3, 7, 36]. This was exemplified by the development of a severe form of cytauxzoonosis in a control cat in Study #2, following infestations with wild-caught ticks from an area of Oklahoma where *A. americanum* are known to carry *C. felis* [29, 34]. Wild felids like bobcats and domestic cats that survive cytauxzoonosis are known to be reservoirs, emphasizing the importance of controlling *A. americanum* in areas populated by wild felids [8, 34]. None of the cats treated with the novel formulation developed any cytauxzoonosis signs, like after three monthly treatments with another isoxazoline topical formulation for cats (sarolaner and selamectin) with comparable efficacy 72 h after infestations with *A. americanum* from the same region [33]. This event illustrates the risk of disease transmission from wild caught ticks to cats, and that laboratory-raised ticks are preferable, when possible.

Cats are also susceptible to infections by a broad range of nematodes and cestodes [22, 23, 28]. The control of multiple and various concurrent parasitic infestations by a broad range of cat parasites is important for cats but also for public health [12, 13, 47]. This combination of esafoxolaner, eprinomectin and praziquantel was approved by the European Medicines Agency on January 13, 2021 with indications covering a broad spectrum of efficacy against the main ecto- and endoparasites of cats [18, 31, 43–46].

Esafoxolaner is the purified and active (S)-enantiomer of afoxolaner, the racemic mixture. The use of a purified enantiomer enables lowering of the exposure dose and thus the potential for side effects and interactions with the other active substances of the combination product.

## Conclusion

In two experimental studies of induced infestations with *A. americanum*, NexGard^®^ Combo provided a high level of efficacy in the treatment and control of *A. americanum* tick infestations for at least one month.

## Competing interest

The work reported herein was funded by Boehringer-Ingelheim. Some of the authors are current employees of Boehringer-Ingelheim Animal Health. Other than that, the authors declare no conflict of interest. This document is provided for scientific purposes only. Any reference to a brand or trademark herein is for information purposes only and is not intended for any commercial purposes or to dilute the rights of the respective owners of the brand(s) or trademark(s).

NexGard^®^ is a registered trademark of the Boehringer-Ingelheim Group.
